# Scientific Validation and Clinical Application of Lung Cancer Organoids

**DOI:** 10.3390/cells10113012

**Published:** 2021-11-04

**Authors:** Dahye Lee, Yoonjoo Kim, Chaeuk Chung

**Affiliations:** 1 Division of Pulmonology and Critical Care Medicine, Department of Internal Medicine, College of Medicine, Chungnam National University, Daejeon 34134, Korea; ziczi02@naver.com (D.L.); misspims@naver.com (Y.K.); 2 Infection Control Convergence Research Center, Chungnam National University School of Medicine, Daejeon 35015, Korea

**Keywords:** lung neoplasms, organoids, culture media, coculture techniques, clinical study

## Abstract

Lung cancer organoid (LCO) is a novel model of lung cancer that facilitates drug screening. However, the success rate of LCOs varies from 7% to 87%, and the culture medium compositions are markedly different. Airway organoid media can be used for LCO cultures, but this promotes the overgrowth of normal cell organoids especially in LCOs from intrapulmonary lesions. Several modified media are specifically utilized for promoting the cancer cell’s growth. For culturing high-purity LCOs, cancer cells from metastatic lesions and malignant effusions are used. Recently, single-cell RNA sequencing has identified previously unknown cell populations in the lungs and lung cancer. This sequencing technology can be used to validate whether the LCO recapitulates the heterogeneity and functional hierarchy of the primary tumor. Several groups have attempted to culture LCOs with mesenchymal cells and immune cells to recapitulate the tumor microenvironment. Disease modeling using LCO provides novel insight into the pathophysiology of lung cancer and enables high-throughput screening for drug discovery and prognosis prediction. An LCO model would help to identify new concepts as a basis for lung cancer targeting by discovering innovative therapeutic targets.

## 1. Introduction

Despite innovations in targeted therapy and immunotherapy in the last decade, lung cancer remains the most common cause of cancer related death [1,2,3] Tumor heterogeneity and drug resistance hamper the treatment of lung cancer [4]. To identify novel strategies to overcome these problems, faithful lung cancer models are needed for preclinical research.

Organoids are three-dimensional cellular complexes that originate from embryonic stem cells, induced pluripotent stem (iPS) cells, or adult stem/progenitor cells [5,6,7,8]. They mimic the basic structure and function of the primary organ. Organoids are composed of several cell populations whereas spheroids are simple collections of one cell type [6,7]. Organoid culture has opened new avenues for both basic and translational medicine. Using an organoid model, disease pathophysiology can be investigated, and novel treatment strategies can be evaluated.

Cancer organoids show promise for cancer research by recapitulating the tumor characteristics and heterogeneity [9]. The use of lung cancer organoids (LCOs) enable reliable high-throughput drug screening [10,11,12]. However, the success rate of LCO culture, the culture medium composition, and tissue processing differ among studies. Because the overgrowth of normal lung organoids is common in LCOs originating from surgical specimens, tumor cell purity is a critical issue for studies using LCOs. There is still controversy as to whether LCOs reflect tumor heterogeneity. Therefore, research aiming to improve LCO purity and validate the recapitulation of primary tumors is underway.

Here, we review recent studies of LCOs and discuss culture media, tissue source, and processing. We also address co-culture, single-cell RNA sequencing, disease modeling, and clinical applications.

## 2. Lung Organoids

### 2.1. Lung Stem Cell, Development, and Mesenchymal Cell

Lung organoids can be divided into airway and alveolar organoids [13,14,15]. Airway organoids originate from basal cells [8,16], which proliferate and differentiate to ciliated, goblet, and club cells in cyst-like structures. Alveolar organoids comprise alveolar type II cells (AT2), which differentiate to AT1 cells and produce bud-shaped alveolar organoids [17]. Formation of lung organoids partly recapitulates lung development in which bone morphogenic protein (BMP)-4, fibroblast growth factor (FGF)-7/10, and Wnt/β-catenin play important roles [18,19]. In vivo, epithelial cells such as basal and AT2 cells closely interact with adjacent mesenchymal cells for proliferation and differentiation [20,21]. A receptome and secretome study showed that FGF-7, BMP, and IL-6 are vital for these interactions [18] (Table 1).

### 2.2. Organoid Culture Media

Medium composition is a critical determinant of the success of lung organoid culture. Generally, lung organoid culture media contain several factors that are crucial for lung development and the interaction between epithelial cells and mesenchymal cells [24]. FGF7, FGF10, Wnt/β-catenin, and BMP inhibitors are added to lung organoid media [19,25]. In addition, EGF and inhibitors of TGF-β can be added for organoid size control and suppression of epithelial–mesenchymal transition (EMT), respectively [26]. To prevent anoikis of single cells and increase the cell survival, the ROCK inhibitor, Y-27632, is also used for several days after the initial seeding or passage (Table 1).

The medium compositions of normal airway and alveolar organoids are similar (Table 1). An important difference is WNT signaling, the activation of which is essential for clonal expansion of AT2 cells during alveolar organoid development. However, airway organoids express WNT, so strong WNT signaling impairs the formation of airway organoids [16,23]. To stimulate WNT signaling, WNT conditioned medium or the GSK3β inhibitor, CHIR99021, is commonly used. WNT-conditioned medium from L-Wnt3A cells can show significant inter-batch differences in WNT activity. R-spondin1 conditioned medium supports WNT signaling by stabilizing the cytosolic β-catenin level [15,16,27]. Ebisudani et al. introduced serum-free Afamin-Wnt 3A containing culture medium for the long-term culture of alveolospheres [15].

The BMP inhibitor Noggin increases the number of stem cells and blocks their differentiation. FGF7 and FGF10 produce large organoids and induce organoid branching. EGF is not essential for organoid formation, but it increases the size of alveolar organoids [16,22]. SB431542 and A83-01 inhibit TGF-β signaling and lengthen organoid growth [13,14]. In addition, SB202190, a p38 inhibitor, is used to overcome organoid growth arrest (Table 1).

### 2.3. Clinical Application of Lung Organoids

Lung organoids have promoted research on pulmonary diseases, including cystic fibrosis (CF). Genetic modification of the cystic fibrosis transmembrane conductance regulator gene in lung organoids provided insight into CF pathogenesis. Forskolin-induced swelling tests with intestinal or lung organoids enable diagnosis and personalized treatment of CF [16,22].

Lung organoids have also been used as a model for bacterial and viral infection. For example, immunologic reactions and the microbial life cycle were investigated by infecting lung organoids with Pseudomonas aeruginosa or Cryptosporidium [28]. Recently, lung organoids have been used in SARS-CoV-2 research [14,27] to investigate the route of infection and the immunological reactions of infected cells.

Lung organoids are also used to study lung regeneration. Recent single-cell analysis of lung-regeneration-associated organoid models revealed an intermittent cell population that transits from type II to type I pneumocytes [29]. They were named damage-associated transient progenitors, pre-alveolar type 1 transitional cell states, and alveolar differentiation intermediate [14,30,31].

## 3. Lung Cancer Organoids

### 3.1. Accomplishments and Challenges of LCO

LCOs can be cultured with high success rates and facilitate high-throughput drug screening. However, LCOs have various limitations, such as low success rates and normal cell contamination.

Clevers et al. first reported the culture of LCOs in 2018 [32]. They cultured LCOs from four surgically resected tissues and two core-needle biopsy tissues with AO medium containing FGF7, FGF10, Noggin, and SB202190. As expected, the LCOs contained many healthy normal airway organoids. To suppress normal cell growth, they used the MDM2 inhibitor, Nutlin-3a, which inhibits the growth of cells with wild-type p53 [32]. Subsequently, several culture media were developed to increase the purity of cancer cells in LCOs; the success rates of the LCO culture ranged from 7–87% [10,12,16,32,33,34]. LCOs facilitate personalized drug screening and prediction of patient-specific drug responses. However, current LCO culture methods are insufficient to produce high-purity LCOs, especially from intrapulmonary lesions [35] (Table 2).

Additionally, Jeong et al. reported a high success rate of LCOs from small-cell lung cancer (SCLC) tissues using a medium based on EGF and FGF for SCLC-LCO culture. Moreover, the addition of WNT3A or R-spondin-1 enabled the long-term expansion of SCLC organoids [32].

Most LCO studies to date have involved surgical resection specimens. Few studies have cultured LCOs from tissues obtained from distant LN biopsies, percutaneous needle biopsies, and EBUS biopsies [11,12,33,34,36]. Moreover, the culture success rate of LCOs from small biopsy tissue is significantly lower than that of LCOs from surgical tissue [10,33,34,37] (Table 2). Further studies with small biopsy samples are needed for personalized medicine for patients with inoperable lung cancer.

### 3.2. Purity of Cancer Cells in LCOs

Successful LCO culture and establishment of LCO banks have been reported [10,11,12,35,36]. LCOs can be used for selecting personalized medicine and predicting the drug response of patients. Cho et al. showed that LCOs can predict drug responses such as progression-free survival and objective response [39]. LCOs have the potential for high-throughput drug screening [10,11,12]. 

However, the success rate of LCO culture varies depending on studies, and several groups demonstrated very low purity of cancer cells in LCOs [33,35]. The success of LCO culture can be determined by several different factors including organoid formation, long-term expansion, and cancer cell purity. However, the criteria for long-term expansion and the validation methods of cancer cell purity are very different, depending on the study. In culture conditions in which normal cells and cancer cells are mixed, normal lung organoid can eventually dominate depending on the media. Therefore, to culture LCOs with high cancer cell purity, the source of primary tissue and the culture media should be carefully selected.

Jang et al. used the minimum basal medium to suppress the formation of normal lung organoids for LCO culture. The medium contains FGF, N2, B27, and a ROCK inhibitor, but not WNT3a or Noggin. They reported an 87% success rate for LCO culture [10]. Another group used a culture medium containing Noggin, FGF-7, FGF-10, and A83-01, which is similar to airway organoid medium. Interestingly, the success rate of a pure LCO culture was 17% and the organoids from 80% of intrapulmonary lesions were normal lung organoids [35]. Moreover, Nutlin-3a did not exclude normal cells because about 40% of lung cancers harbor wild-type p53 based on Cancer Genome Atlas data.

Yamatsuji et al. generated lung tumoroids in only 7% (3/41) of cases [33]. Among various media, AO medium had the highest LCO culture success rate. Additionally, some LCOs did not grow in any medium, whereas others grew well in all media, including serum-free DMEM/F12 medium [33]. These results emphasize the importance of selecting an appropriate medium based on the characteristics of the primary tumor.

To achieve high-purity LCOs, organoids can be produced from metastatic tissue or malignant effusion, enabling normal lung cells to be excluded. Small biopsy tissues from the tumor core can enhance cancer cell purity in LCOs compared to surgically resected tumors. Cancer organoids were established using cells obtained by fine-needle aspiration of melanoma and thyroid [40]. This method could be applied to the production of LCOs from patients with inoperable lung cancer [40].

Several studies are underway to identify an LCO culture medium that selects for cancer cells and inhibits normal cells [41]; however, the efforts have been hampered by intertumoral and intratumoral heterogeneity.

### 3.3. Validation of LCOs

How closely LCOs recapitulate the characteristics of the original tumor is a critical determinant of the value of LCOs. LCOs have been validated to recapitulate the primary tumor in various ways [10,34,35], such as morphology and histology. In most studies, LCOs represented the original-tumor in H&E staining and immunohistochemistry findings, such as thyroid transcription factor-1, cytokeratin 5/7, and p63 [10,35]. The expression and polarity of p63 can differentiate normal lung organoids from LCOs [10]; LCOs are more compact than normal lung organoids. In some studies, cystic organoids were handpicked for high-purity organoid culture [10,35]. However, judging LCOs based on morphology alone is difficult because LCOs can have a cystic morphology similar to normal lung organoids, which can be of atypical shape after several passages. The genetic characteristics of tumor specimens and matching LCOs were compared by whole-exome sequencing and assessing copy number (CN) variation, variant allele fraction (VAF) distribution, and single nucleotide polymorphisms [10]. CN profiling can identify most samples as tumor or normal, but some tumors retain a normal CN profile [35]. A widely distributed VAF may mean that LCOs consist of a heterogeneous population similar to the tumor but could also suggest that normal cells are present. Differences in genetic profiles between early- and late-passage LCOs should be carefully examined and interpreted because a specific cell population can be selected after long-term LCO culture with particular media. Single-cell RNA sequencing (scRNA-seq) is one of the most precise tools for validating whether LCOs really recapitulate the primary tumor’s characteristics and heterogeneity. 

### 3.4. Tumor Microenvironment and Co-Culture

Cancer cells dynamically interact with adjacent mesenchymal and immune cells. With the advent of immunotherapy, the importance of the tumor microenvironment has increased. Therefore, studies using conventional experiments with cancer cell lines and models with immunocompromised mice have several limitations for immune-oncology studies.

Co-culture of organoids with immune cells or mesenchymal cells can represent the in vivo microenvironment. In a mouse model, lung stem cells including bronchioalveolar stem cells (BASCs) and AT2 cells can be cultured with mesenchymal cells. Interactions with mesenchymal cells affect the differentiation and stemness of BASCs and AT2 cells [12,20,21,42]. For example, BASCs differentiate into alveolar lineages when cultured with endothelial cells [21], and type II cells produce alveolar spheres when cultured with fibroblasts [39]. Co-culture of type II pneumocytes and fibroblasts revealed that Wnt signaling maintains AT2 stemness and prevents differentiation to AT1 cells [43] (Table 3). Human AT2 cells form alveolospheres with or without support from fetal lung fibroblasts [23,40]. However, co-culture of human AT2 cells with adult human lung mesenchymal cells induced formation of bronchiolar organoids [44,45].

Culture of mouse tumor organoids with fibroblasts, lymphocytes, or macrophages showed that stromal cells and tumor-infiltrating lymphocytes (TILs) within tumor organoids recapitulate the tumor immune and fibroblastic repertoire [48]. IL-2 supported the growth of intra-organoid CD3+ TIL, CD4+, and CD8+ cells for about 1 week [48].

There are few reports of the culture of human LCOs with mesenchymal or immune cells. Krijin et al. cultured LCOs and peripheral blood mononuclear cells to test the effect of immunotherapy [33]. They activated PD-L1 in LCOs by IFN-γ and supported T-cell proliferation with IL-2. They co-cultured LCOs and CD8+ T cells for 2 weeks and evaluated the expansion of tumor-reactive T cells [33]. Technical issues including immune reactions and lack of a medium that supports both cell types (Table 3) hamper the co-culture of LCOs and immune cells.

Effort is being devoted to recapitulating the tumor microenvironment in LCO models and determining the effect of immune-checkpoint blockade. This co-culture system could enable not only expansion and selection of chimeric antigen receptor T (CAR T) cells but also neoantigen reactivity screening [43].

### 3.5. Single-Cell RNA Sequencing of LCOs

scRNA-seq of the human lung revealed 14 previously unknown cell populations. scRNA-seq of lung adenocarcinoma patients showed the subtypes of cancer cells and changes in the immunosuppressive microenvironment [37]. These data provide insight into molecular and cellular profiles and enable identification of potential targets for lung cancer diagnosis and treatment.

Tumor organoids have been used for drug screening for personalized medicine. However, tumor organoid models have not been fully validated in terms of whether they reflect tumor cellular heterogeneity [37]. Since bulk RNA sequencing can show only the average characteristics of tumors, there is a limit to validating heterogeneity. scRNA-seq revealed that pancreatic duct adenocarcinoma (PDAC) organoids comprise multiple cell types, including cyclic cell clusters, differentiating cell clusters, and cytokine-secreting cell clusters [49]. These data confirm that the functional hierarchy is conserved in PDAC organoids.

No scRNA-seq study of lung cancer organoids has yet been published. Jang et al. analyzed the VAF distribution in LCOs and original tumor tissue. The VAF value in LCOs was 0.1 to 1.0, suggesting that LCOs preserve the heterogeneous cell populations of primary tumors [10]. scRNA-seq of LCOs will validate whether they recapitulate the spatiotemporal heterogeneity and hierarchy of lung cancer. Therefore, LCOs can be used for in-depth studies of tumor evolution and targeting of chemical-resistant cell populations.

### 3.6. Disease Modeling and Clinical Application of LCOs

Hitherto, several lung cancer models including lung cancer cell lines, primary cell culture, and patient-derived xenograft models have been used to recapitulate human lung cancer [7]. In addition, genetically engineered mouse models with Kras^G12D^, p53, EGFR, EML4-ALK fusion kinase, or ROS fusion kinase have been used for lung cancer research [50,51,52,53,54]. However, each has advantages and disadvantages, so a cost-effective human-tissue model that adequately recapitulates the tumor characteristics is needed.

In terms of success rate, generation time, genetic manipulation, and cost, LCO is a valuable tool for drug screening and predicting the drug response of patients. Organoid models can be used to detect rare genetic mutations and optimize treatment drugs [10,39] (Table 4). Kim produced LCOs from KrasG12D or KrasG12D, p53fl/fl mice [54]. They also differentiated iPS to type II pneumocytes, and overexpressed Kras in the alveolar organoids. Kras-overexpressing mouse or human alveolar organoids lost AT2 cell differentiation markers and recapitulated the characteristics of early lung cancer [54]. A microwell array chip with LCOs enabled prediction of drug responses within 1 week [11]. Tissue was processed by only mechanical chopping and a strainer [11]. This rapid drug screening will enable personalized medicine for lung cancer. Several clinical studies using patient-derived LCOs are aiming to determine whether LCO models predict the prognosis and drug responses of lung cancer. In addition, co-culture of tumor-infiltrating lymphocytes with LCOs is under investigation to screen T-cell responses (Table 4).

## 4. Conclusions

LCOs have considerable potential as a model of lung cancer. However, issues related to the purity of cancer cells and the composition of LCO media remain to be overcome. Insight into the cellular heterogeneity of LCOs would enable their validation and increase their purity. Co-culture of LCOs with mesenchymal cells and immune cells will enable screening for immuno-oncology drugs and CAR T cells. Established LCO models will help in identifying new concepts as a basis for lung cancer targeting and enable discovery of novel therapeutic targets.

## Figures and Tables

**Table 1:** Human lung organoid culture media.

Organoid (Reference)	Isolated Cell	WNT Signaling			BMP Inhibitor	FGF		EGF	TGFβ Inhibitor	ALK Inhibitor	p38 MAPKi	ROCK Inhibitor	Supplement	Etc.
		WNT 3A	CHIR 99021	R-Spondin	Noggin	FGF 7	FGF 10		A83-01	SB 431542	SB 202190	Y-27632	NA, B27, NAC	
Airway organoid [16]	Lung epithelial cells													
Alveolar organoid [22]	Embryonic lung epithelial tip cells													
Alveolosphere [14]	AT 2 cells (HTII-280^+^ cells)										BIRB-786			Heparin, IL-1β
Distal lung organoid [13]	Lung epithelial cells (EPCAM^+^ cells)													
Alveolosphere [15]	AT 2 cells (HTII-280^+^ cells)	Afamin-Wnt-3A												FGF-2, IGF-1
3D alveolar stem cell culture [23]	AT 2 cells (HTII-280^+^ cells)													

NA: nicotinamide, NAC: N-acetylcysteine, MAPKi: MAPK inhibitor, IGF-1: insulin like growth factor, FGF: fibroblast growth factor.

**Table 2:** Studies of lung cancer organoid.

P.I. (Year)	Cancer Type and Origin	Success Rate [Validation Methods]	Culture Media or Remarks	Reference
Inoue (2013)	Lung cancer cells	80%	Embryonic stem cell culture media, Plus FGF2	[38]
Voest (2018)	AC (*n* = 3), SQC (*n* = 2), NOS (*n* = 1) Biopsy (*n* = 2), resection (*n* = 4)	NSCLC organoid from 6 patients One sample contained normal airway organoid	AO media containing R-spondin1, FGF7, FGF10, Noggin, A83-01, SB202109, B27, NAC, and NA	[33]
Clevers (2019)	AD, SQC, LCNEC (*n* = 34) Biopsy of metastatic lesion, resection	Resection: 88% (*n* = 16): normal tissue contained. Biopsy: 28% (*n* = 18) Orthotopic transplantation: 30% (*n* = 12) (morphology, histology, whole-genome sequencing)	AO media: 5uM Nutlin-3a	[16]
Jang (2019)	AD, SQC, ASC, LCC, SCLC (*n* = 23) biopsy, resection	Long term expansion (>6 months) : 87% (*n* = 20) (SNP genotype, VAF distribution)	MBM containing basic FGF, N2, B27, ROCK inhibitor (deletion of Wnt3a and noggin)	[10]
Voest (2020)	AC, SQC (*n* = 58), biopsy (*n* = 30), resection (*n* = 28),	Overall: 17% (*n* = 9), resection: 18% (*n* = 5) Biopsy: 13% (*n* = 4) (Copy number profile, IHC)	Media containing Noggin, FGF-7, FGF-10, A83-01, SB202190, 5 μM Nutlin-3a	[36]
Tsao (2020)	AD (*n* = 19), SQC (*n* = 15), AD-PDX (*n* = 16), SQC-PDX (*n* = 26)	Short-term culture (1–3 months, 1–9 passages): 72% (*n* = 47) Long-term culture (>3 months, >10 passages): 15% (*n* = 10) (Whole-exome and RNA sequencing)	M26 containing CHIR 99021, A83-01, EGF, FGF-4, FGF-10, SAG	[12]
Yamatsuji (2021)	AD (*n* = 29), SQC (*n* = 7), ACIS (*n* = 1), SCLC(*n* = 2), PC (*n* = 2)	Long term culture (>13 months, >36 passages) Overall: 7% (*n* = 3), Primary tumor 3.6% (*n* = 1) LN: 100% (*n* = 1), ME: 50% (*n* = 1) (Karyotyping of chromosomes)	AO media was superior to 3 different media (media of Jang, Tsao’s, and Inoue’s groups)	[35]
Cho (2021)	Advanced AD (*n* = 100)	83.0% (*n* = 83), ME (*n* = 77), Brain metastasis (*n* = 3), Bone metastasis (*n* = 1), lung primary tumor (*n* = 2) (Whole-exome, RNA sequencing)	AO media	[39]
Jeong (2021)	SCLC (*n* = 10)	Long-term expansion (>9 months) 80% (*n* = 8) (Morphology, molecular characteristics, genomic profile)	EGF, FGF-based media ± WNT3A or R-spondin-1	[34]

P.I.: principal investigator, AC: adenocarcinoma, SQC: squamous cell carcinoma, ASC: adenosquamous cell carcinoma, LCC: large-cell carcinoma, SCLC: small-cell lung cancer, AO: airway organoid, PB: peripheral blood, LCNEC: large-cell neuroendocrine carcinoma, MBM: minimum basal media, PD: pleomorphic carcinoma, ACIS: adenocarcinoma in situ, ME: malignant effusion, PDX: patient-derived xenograft, SAG: smoothened ligand. SNP: single nucleotide polymorphism, VAF: variant allele frequency, IHC: immunohistochemistry.

**Table 3:** Co-culture with organoids from mouse adult stem cells.

Lung Epithelial Cell	Co-Culture	Effect	Reference
Mouse airway basal stem cell (ABSC)	Lung fibroblast, endothelial cell	Mesenchymal cells Influence ABSC’s proliferation and differentiation in vitro	[42]
Mouse bronchioalveolar stem cell (BASC)	Endothelial cell	BASC differentiates to alveolar lineage	[21]
Mouse AEC2 (HT-280^+^ cell)	PDGFRα^+^ stromal cells	AEC2s self-renewal and differentiate to AEC1s, forming alveolosphere	[40]
Mouse BASC	Lung-resident mesenchymal cells	BASC forms bronchioalveolar lung organoid (BALO) that express markers of airway and alveoli	[46]
Mouse AEC2	Fibroblast	Wnt-secreting fibroblasts maintain AT2 stemness and prevents differentiation to AT1	[44]
Human basal cell	Fibroblast	Basal cell forms tracheospheres containing basal, ciliated, and mucosecretory cells	[47]
Human lung cancer PC9 cell	Podoplanin^+^ cancer-associated fibroblast (CAF)	With CAFs, PC9 cell form cancer organoid CAF promotes cancer cell growth	[37]
Human lung cancer organoid	Lung cancer organoid	Test the effect of immunotherapy	[33]

**Table 4:** Clinical trials that utilized lung cancer organoid.

Topic of Study	Study Type Phase	Outcome Measures	Status/Location	Reference
Prospective primary human lung cancer organoids to predict treatment response	Observational, prospective	Biobanking of lung cancer organoid PDX models of lung cancer Tumor response	Recruiting/Zuyderland Medical Center, The Netherlands	NCT04859166
Patient-derived organoid model and circulating tumor cells for predicting treatment response of lung cancer	Observational, prospective	Biobanking of patient-derived organoid Correlation of PDO and circulating tumor cell	Recruiting/M.D. Anderson Cancer Center, the United States	NCT03655015
Drug sensitivity correlation between patient-derived organoid model and clinical response in NSCLC patients	Observational, cross-sectional	Correlation of ex vivo sensitivity test on patient derived organoid models	Unknown/People’s Hospital of Hebei, Province, China	NCT03453307
TCR-T cell for immunotherapy of lung cancer	Phase 1	Coculture of organoid and TIL will be utilized to screen tumor responsive T cell	Recruiting/Hospital of Guangzhou Medical University, China	NCT03778814
High-dose vitamin C intravenous infusion in patients with solid tumor	Phase 2	3 month DCR, in vitro activity of vitamin C in tumor organoids	Recruiting/New York-Presbyterian Hospital, the United States	NCT03146962

DCR: disease control rate, PDO: patient-derived organoid, TCR-T cell: T cell receptor engineering T cell.

## Data Availability

Not applicable.
